# Stroke disability burden and rehabilitation service-capacity proxies in China, 1990–2023: A population-based analysis using GBD 2023 and national service statistics

**DOI:** 10.1371/journal.pone.0361758

**Published:** 2026-11-06

**Authors:** Yumin Zhou, Wenke Fan, Dandan Lv, Qian Li, Yu Cao, Hao Tian, Weiwei Guo, Xia Bi

**Affiliations:** 1 Shanghai University of Medicine and Health Sciences Affiliated Zhoupu Hospital, Shanghai, China; 2 Department of Rehabilitation Medicine, Huashan Hospital, Fudan University, Shanghai, China; University of the Witwatersrand Johannesburg, SOUTH AFRICA

## Abstract

China’s stroke burden is increasingly shaped by survival with disability, population ageing, and demand for rehabilitation and long-term support. Yet the extent to which stroke disability burden has shifted towards older-old and oldest-old adults, and how this burden relates to community and elder-care service capacity, remains insufficiently quantified. We conducted a population-based secondary analysis of Global Burden of Disease Study 2023 estimates for China from 1990 to 2023, focusing on ischaemic stroke and intracerebral haemorrhage. We examined disability-adjusted life years (DALYs), years lived with disability (YLDs), YLD share of DALYs, age-specific patterns among adults aged 50 years or older, estimated annual percentage changes (EAPCs), and three-factor decomposition of absolute DALY changes. We also linked 2023 stroke YLDs with national community health and elder-care service indicators as descriptive capacity proxies. From 1990 to 2023, the YLD share of DALYs increased from 10.5% to 22.3% for ischaemic stroke and from 1.8% to 3.3% for intracerebral haemorrhage. The crude all-age ischaemic stroke YLD rate increased by 2.75% per year (95% CI 2.68 to 2.82), while the crude all-age intracerebral haemorrhage DALY rate declined but its YLD rate and YLD share increased. Crude all-age rates were interpreted as population-level burden indicators influenced by population age structure, alongside age-stratified and decomposition analyses. Among adults aged 50 years or older, disability burden shifted towards older-old and oldest-old groups, particularly for ischaemic stroke. In 2023, combined all-age YLDs from ischaemic stroke and intracerebral haemorrhage were approximately 4.77 million, corresponding descriptively to 128.2 YLDs per community health service centre/station and 16.7 YLDs per community health bed. Stroke in China is increasingly a survivorship and disability challenge. The burden-to-capacity proxy indicators provide planning context but do not directly measure rehabilitation access, adequacy, quality, or unmet need.

## Introduction

Stroke remains a leading cause of death and disability worldwide, but the public health meaning of stroke burden is changing as acute care improves and more people survive with chronic impairment. For health systems, this shift requires attention not only to mortality, incidence, and total disability-adjusted life years (DALYs), but also to years lived with disability (YLDs), rehabilitation demand, functional dependence, and long-term support [1–4].

In China, this survival-to-survivorship transition is occurring alongside rapid population ageing and continuing epidemiological transition. The number of older stroke survivors is expected to increase, and the clinical profile of post-stroke disability is likely to become more complex as survivors increasingly have frailty, multimorbidity, cognitive impairment, sarcopenia, pre-existing disability, and caregiver dependence [5–7]. These features are particularly relevant for adults aged 75 years or older and 85 years or older, for whom rehabilitation goals often extend beyond impairment recovery to maintaining independence, preventing avoidable institutionalisation, and supporting families.

Conventional stroke burden studies have provided important evidence on mortality, incidence, DALYs, risk factors, and temporal trends. However, they may not fully capture the structure of rehabilitation-relevant burden. YLDs reflect non-fatal disabling outcomes and therefore provide a closer proxy for the scale of rehabilitation and long-term functional support needs than mortality-based indicators alone. Similarly, the proportion of DALYs attributable to YLDs, referred to here as YLD share, can indicate whether the burden is shifting from premature mortality towards chronic disability among survivors [8–14].

A second knowledge gap concerns the connection between epidemiological burden and service-planning context. Population-level estimates of stroke disability are often reported without reference to community health services, rehabilitation resources, or elder-care infrastructure. Yet in practice, post-stroke care depends on coordination across hospitals, community health services, home-based care, and long-term-care facilities. Descriptive burden-to-capacity indicators cannot measure service adequacy, access, unmet need, or quality directly, but they can place epidemiological estimates alongside selected national service-resource denominators for planning interpretation.

We therefore used Global Burden of Disease Study 2023 estimates to quantify rehabilitation-relevant stroke burden in China from 1990 to 2023. We focused on ischaemic stroke and intracerebral haemorrhage because these major subtypes differ in survival, disability profile, and long-term care needs. The objectives were to describe temporal trends in DALYs and YLDs, quantify changes in YLD share, evaluate shifts towards older-old and oldest-old adults, decompose changes in absolute burden into demographic and rate components, and contextualise 2023 stroke disability burden against national community health and elder-care service capacity proxies. Unlike a conventional descriptive burden update, this analysis reframes stroke burden as a survivorship, rehabilitation, and long-term-care planning problem in an ageing society.

## Methods

### Study design and data sources

We conducted a population-based secondary analysis of publicly available aggregate estimates from the Global Burden of Disease Study 2023 for China from 1990 to 2023. The study was designed to quantify rehabilitation-relevant stroke burden and its relationship with selected service-capacity proxies rather than to provide a comprehensive update of stroke epidemiology. The GBD framework provides standardised estimates of disease burden across countries, causes, age groups, sexes, and years using a unified analytical approach [15].

We analysed two stroke subtypes: ischaemic stroke and intracerebral haemorrhage. Estimates were extracted from the IHME GBD Results Tool using the predefined GBD cause names ‘Ischemic stroke’ and ‘Intracerebral hemorrhage,’ following the GBD cause hierarchy. ICD-9 and ICD-10 reference mappings are provided in S1 Table for transparency, but cause assignment in this study was based on GBD cause categories rather than manual aggregation of diagnosis codes.

### Outcomes and rehabilitation-relevant indicators

The main outcomes were DALYs and YLDs. For each subtype and year, we extracted absolute numbers and rates per 100,000 population. The all-age DALY and YLD rates reported in this study are crude rates rather than age-standardized rates; they reflect both age-specific burden and the population age structure. We retained these crude all-age rates because they describe population-level burden relevant to service-planning pressure, but they should not be interpreted as age-independent epidemiological trends. Absolute DALYs and YLDs were used to describe the scale of burden. To characterise the disability component of stroke burden, we calculated YLD share as YLDs divided by total DALYs for each subtype and year.

We treated YLDs and YLD share as rehabilitation-relevant indicators because they describe non-fatal disability burden. These indicators do not measure rehabilitation use, eligibility, functional status, or quality of care directly, and they should therefore be interpreted as burden indicators rather than direct measures of service demand.

### Age-stratified analysis

Age-stratified estimates were analysed among adults aged 50 years or older in 5-year age bands from 50–54 years to 95 years or older. We first compared burden in adults younger than 65 years and 65 years or older to maintain comparability with conventional older-adult analyses. We then constructed clinically relevant older-age subgroups of 50–64, 65–74, 75–84, and 85 years or older to evaluate whether rehabilitation-relevant burden shifted towards older-old and oldest-old populations. For each subgroup, we calculated DALYs, YLDs, YLD share, DALY rate, and YLD rate.

### Decomposition analysis

We used a three-factor decomposition approach to assess the contributions of population growth, population ageing, and age-specific DALY rate change to changes in absolute DALYs between 1990 and 2023 [16,17]. Because the age-stratified estimates used in this analysis covered adults aged 50 years or older, decomposition was conducted within the 50 + age-stratified dataset rather than the all-age dataset.

Age-specific population counts were reconstructed by dividing age-specific DALY numbers by corresponding DALY rates and multiplying by 100,000. We then used 1990 and 2023 total population size, age composition, and age-specific DALY rates to construct counterfactual burden estimates. Positive components indicate factors that increased absolute burden, whereas negative components indicate factors that reduced absolute burden.

### Trend analysis

To assess whether changes reflected sustained trends rather than endpoint differences alone, we estimated EAPCs for annual crude all-age DALY rates, crude all-age YLD rates, and YLD share from 1990 to 2023. For each indicator, we fitted a log-linear regression model: ln(indicator) = alpha + beta × year. EAPC was calculated as 100 × [exp(beta) – 1], with 95% confidence intervals calculated as 100 × [exp(beta ± 1.96 × SE(beta)) – 1], where SE(beta) is the standard error of beta. These EAPCs describe trends in the observed crude population-level rates and therefore remain influenced by changes in population age structure.

### Service-capacity proxy analysis

To contextualise rehabilitation-relevant burden in relation to service-planning capacity, we linked 2023 stroke YLD estimates with national service-resource indicators from official Chinese health and ageing statistics. Denominators were taken from the 2023 Statistical Bulletin on China’s Health Development, the 2023 National Bulletin on the Development of Ageing, and the China Health Statistics Yearbook 2024, using year-end 2023 values and exact yearbook table values where available [18–20].

We selected community health service centres/stations, community health personnel, community health beds, elder-care beds, and nursing-type elder-care beds as service-capacity proxies because post-stroke rehabilitation and long-term functional support commonly require community-based health services and age-care infrastructure. We calculated descriptive burden-to-capacity ratios by dividing combined YLDs from ischaemic stroke and intracerebral haemorrhage by each denominator. These ratios were intended to support planning interpretation and should not be interpreted as direct measures of stroke-specific rehabilitation access, staffing adequacy, therapy intensity, utilisation, quality, or unmet need. Exact denominator definitions and calculations are provided in S2 Table.

### Statistical analysis and reproducibility

All analyses were descriptive and were conducted using custom-written scripts. The decomposition analysis was implemented using custom code based on the methods described by Kitagawa and Das Gupta rather than a dedicated decomposition package. No individual-level inferential testing was performed because the study used aggregate modelled estimates. Confidence intervals for EAPCs reflect uncertainty in the fitted log-linear trend and should not be interpreted as individual-level statistical inference. GBD uncertainty intervals were retained for directly extracted rates where available. Formal uncertainty propagation was not performed for derived YLD shares, decomposition components, or burden-to-capacity proxy ratios because posterior draws and covariance information across component estimates were not available in the exported dataset; these derived indicators are therefore presented descriptively as point estimates.

For reproducibility, the extracted analytical dataset and statistical code are provided as Supporting Information files. S1 Data contains the cleaned analytical dataset used to reproduce all tables and figures, and S1 Code contains the statistical code used for EAPC, decomposition, and service-capacity proxy calculations.

### Ethics statement

This study used publicly available, de-identified aggregate estimates and public national statistical indicators. No individual-level data, patient contact, human tissue, identifiable personal information, or animal data were involved. Institutional ethics approval and informed consent were therefore not required.

## Results

### Increasing disability share of stroke burden

The disability component of stroke burden increased for both subtypes, but the increase was much larger for ischaemic stroke. In 1990, YLDs accounted for 10.5% of total ischaemic stroke DALYs; by 2023, this proportion had increased to 22.3%, an increase of 11.8 percentage points. For intracerebral haemorrhage, YLD share increased from 1.8% to 3.3%, an increase of 1.5 percentage points (Table 1, Fig 1).

**Table 1:** Rehabilitation-relevant stroke burden indicators, 1990 and 2023.

Stroke subtype	YLDs as % of DALYs, 1990	YLDs as % of DALYs, 2023	Change in YLD share, pp	DALYs number, 1990(million)	DALYs number, 2023(million)	Change in DALYs number, %	Crude all-age DALY rate, 1990(95% UI)	Crude all-age DALY rate, 2023(95% UI)	Change in crude all-age DALY rate, %
Ischaemic stroke	10.5	22.3	11.8	13.38	18.00	34.5	1134.6 (900.6 to 1399.0)	1258.2 (1063.9 to 1441.2)	10.9
Intracerebral haemorrhage	1.8	3.3	1.5	30.57	22.36	−26.8	2592.3 (2058.5 to 3227.7)	1563.3 (1350.7 to 1767.0)	−39.7

DALYs = disability-adjusted life years; YLDs = years lived with disability; UI = uncertainty interval; pp = percentage points. Rates are crude all-age rates per 100,000 population and are influenced by population age structure. YLD share = YLDs/total DALYs. Formal uncertainty propagation was not performed for YLD share.

**Fig 1:**
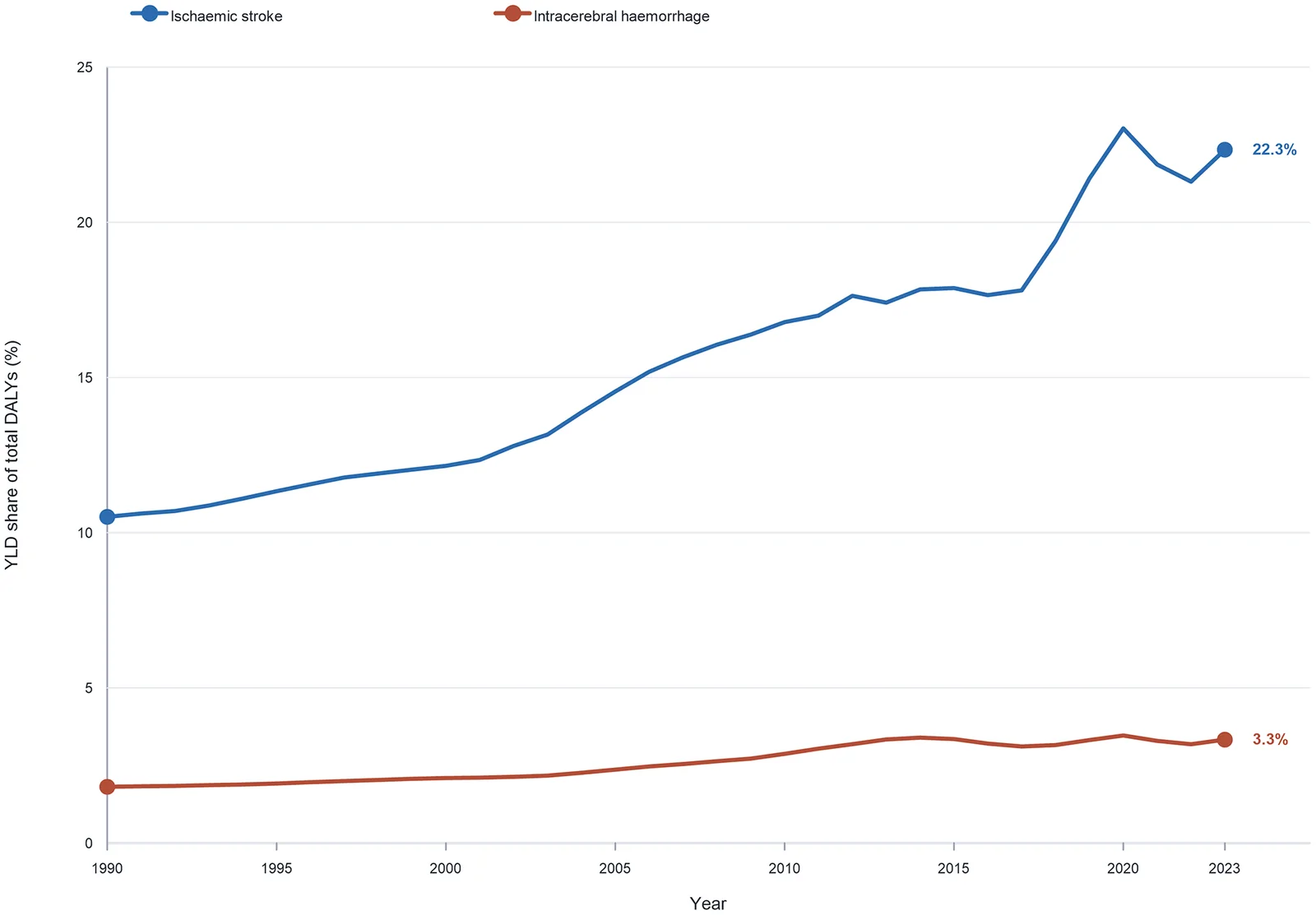
Temporal increase in the disability share of stroke burden in China, 1990–2023.

Lines show the percentage of total DALYs attributable to YLDs for ischaemic stroke and intracerebral haemorrhage. The rising YLD share indicates an increasing contribution of non-fatal disability to overall stroke burden. Point estimates are shown; formal uncertainty propagation for YLD share was not performed.

Both the absolute number and the rate of YLDs increased over time for ischaemic stroke. Intracerebral haemorrhage showed more modest increases in YLDs despite a large reduction in the crude all-age DALY rate. These findings suggest that the structure of stroke burden in China has increasingly shifted towards survivorship with disability, especially after ischaemic stroke.

### Sustained annual trends in rehabilitation-relevant indicators

EAPC analysis supported the interpretation that the disability component of stroke burden increased over the full study period. For ischaemic stroke, the crude all-age DALY rate increased modestly (EAPC 0.27%, 95% CI 0.15 to 0.40), while the crude all-age YLD rate increased more rapidly (EAPC 2.75%, 95% CI 2.68 to 2.82). YLD share also increased (EAPC 2.47%, 95% CI 2.33 to 2.61).

For intracerebral haemorrhage, the crude all-age DALY rate declined (EAPC −1.84%, 95% CI −2.03 to −1.65), but the crude all-age YLD rate still increased (EAPC 0.40%, 95% CI 0.27 to 0.53), and YLD share rose steadily (EAPC 2.28%, 95% CI 2.07 to 2.50) (Table 2).

**Table 2:** Estimated annual percentage changes in rehabilitation-relevant stroke burden indicators, 1990-2023.

Stroke subtype	Indicator	EAPC, % (95% CI)
Ischaemic stroke	Crude all-age DALY rate	0.27 (0.15 to 0.40)
Ischaemic stroke	Crude all-age YLD rate	2.75 (2.68 to 2.82)
Ischaemic stroke	YLD share of DALYs	2.47 (2.33 to 2.61)
Intracerebral haemorrhage	Crude all-age DALY rate	−1.84 (−2.03 to −1.65)
Intracerebral haemorrhage	Crude all-age YLD rate	0.40 (0.27 to 0.53)
Intracerebral haemorrhage	YLD share of DALYs	2.28 (2.07 to 2.50)

EAPC = estimated annual percentage change; DALYs = disability-adjusted life years; YLDs = years lived with disability; CI = confidence interval. EAPCs were estimated from annual crude all-age indicator values from 1990 to 2023. These rates are influenced by population age structure; the confidence intervals quantify uncertainty in the fitted log-linear trend.

### Shift towards older-old and oldest-old adults

Stroke burden became increasingly concentrated not only among adults aged 65 years or older, but also in older-old and oldest-old subgroups. Among adults aged 50 years or older, those aged 65 years or older accounted for 71.8% of ischaemic stroke DALYs in 1990 and 79.7% in 2023. For intracerebral haemorrhage, the corresponding proportions were 60.1% and 68.1%.

When older adults were subdivided, the 75–84 and 85 years or older groups showed particularly important shifts in absolute YLD burden. For ischaemic stroke, YLDs in the 85 years or older group increased from 0.03 million in 1990 to 0.33 million in 2023, while DALYs increased from 0.60 million to 2.65 million. YLD share in this age group increased from 5.4% to 12.5%. For intracerebral haemorrhage, DALYs declined in younger older-adult groups but increased in the 85 years or older group from 0.89 million to 2.28 million, with YLDs increasing from 0.01 million to 0.05 million (Table 3, Fig 2). Age-specific DALY rate patterns across the original 5-year age bands are shown in S1 and S2 Figs.

**Table 3:** Stroke burden by older-age subgroup in China, 1990 and 2023.

Subtype	Age group	DALYs, 1990 (million)	DALYs, 2023 (million)	YLDs, 1990 (million)	YLDs, 2023 (million)	YLD share, 1990 (%)	YLD share, 2023 (%)	DALY rate, 2023	YLD rate, 2023
Ischaemic stroke	50-64 years	3.45	3.47	0.38	1.04	10.9	30.0	1092	328
Ischaemic stroke	65-74 years	4.86	5.61	0.40	1.30	8.2	23.2	4083	948
Ischaemic stroke	75-84 years	3.33	5.41	0.23	0.93	7.0	17.1	9137	1566
Ischaemic stroke	85 + years	0.60	2.65	0.03	0.33	5.4	12.5	14546	1821
Intracerebral haemorrhage	50-64 years	10.07	6.36	0.14	0.22	1.3	3.5	2000	71
Intracerebral haemorrhage	65-74 years	9.00	6.19	0.08	0.15	0.9	2.4	4507	109
Intracerebral haemorrhage	75-84 years	5.30	5.08	0.05	0.11	0.9	2.1	8573	179
Intracerebral haemorrhage	85 + years	0.89	2.28	0.01	0.05	1.0	2.0	12502	250

*Values were calculated within the age-stratified dataset for adults aged 50 years or older. Rates are per 100,000 population.*

**Fig 2:**
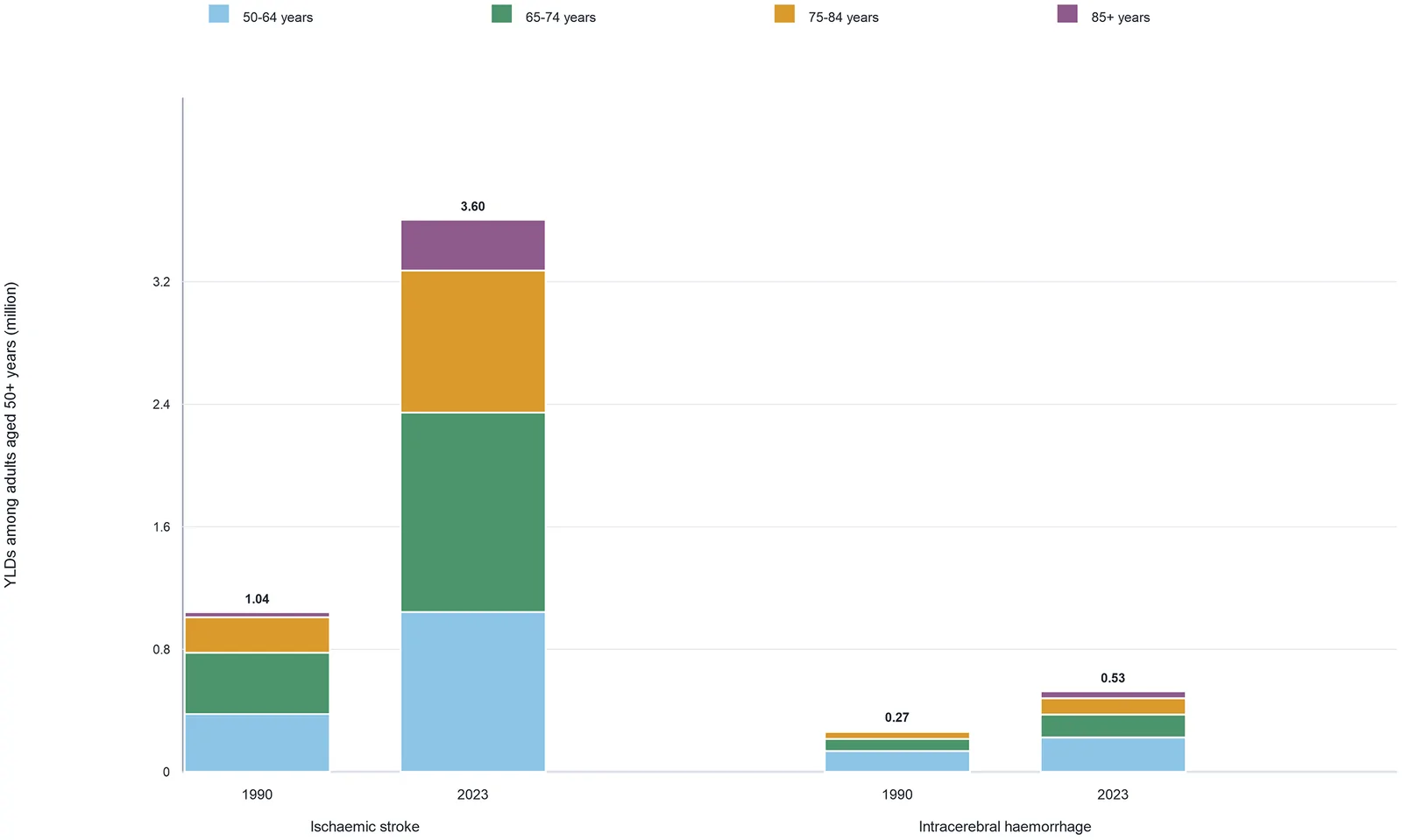
Shift of stroke YLD burden towards older-old and oldest-old adults in China, 1990 and 2023.

Stacked bars show absolute YLDs among adults aged 50 years or older for ischaemic stroke and intracerebral haemorrhage in 1990 and 2023. Age groups are categorised as 50–64, 65–74, 75–84, and 85 years or older. YLDs = years lived with disability.

### Demographic pressure on absolute burden

Decomposition analysis showed that demographic change played a major role in sustaining absolute stroke burden, and that population ageing acted as an independent driver distinct from overall population growth. Within the age-stratified dataset for adults aged 50 years or older, the net change in absolute DALYs for ischaemic stroke from 1990 to 2023 was + 4.915 million. Population growth contributed +21.632 million DALYs, population ageing contributed +5.653 million DALYs, and age-specific DALY rate change contributed −22.369 million DALYs.

For intracerebral haemorrhage, the net change was −5.349 million DALYs. Population growth contributed +44.661 million DALYs, population ageing contributed +7.414 million DALYs, and age-specific DALY rate change contributed −57.424 million DALYs (Fig 3). These findings indicate that favourable age-specific rate changes were partly offset by demographic pressure.

**Fig 3:**
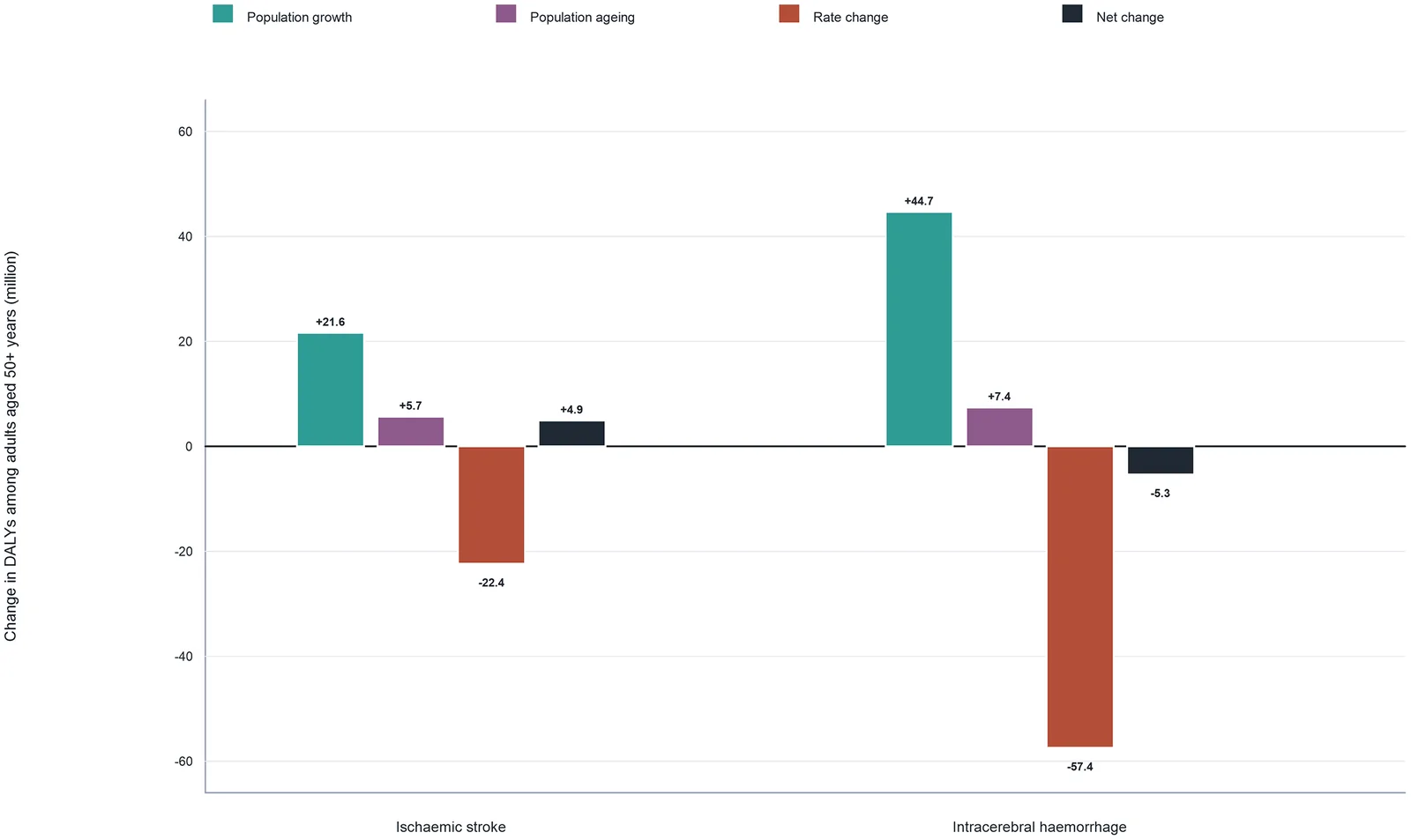
Demographic and rate-change contributions to changes in absolute stroke DALYs, 1990-2023.

Bars show the three-factor decomposition of changes in absolute DALYs among adults aged 50 years or older for ischaemic stroke and intracerebral haemorrhage. Positive values indicate components that increased total DALYs, whereas negative values indicate components that reduced total DALYs. DALYs = disability-adjusted life years. Components are presented as point estimates; formal uncertainty propagation was not performed.

### Divergent long-term trends by stroke subtype

From 1990 to 2023, the crude all-age DALY rate for intracerebral haemorrhage decreased from 2592.3 to 1563.3 per 100,000, a reduction of 39.7%. Over the same period, the corresponding crude all-age rate for ischaemic stroke increased from 1134.6 to 1258.2 per 100,000, an increase of 10.9% (Table 1; S3 Fig). Absolute burden showed the same contrast: ischaemic stroke DALYs increased from 13.38 million to 18.00 million, whereas intracerebral haemorrhage DALYs declined from 30.57 million to 22.36 million.

### Rehabilitation-relevant burden in relation to service-capacity proxies

In 2023, ischaemic stroke and intracerebral haemorrhage together accounted for approximately 4.77 million all-age YLDs in China, including 2.86 million YLDs among adults aged 65 years or older. National service-capacity indicators included 37,177 community health service centres/stations, 778,000 personnel in these centres/stations, and 285,176 community health service beds.

When placed alongside the selected national service-resource denominators, this corresponded descriptively to 128.2 all-age stroke YLDs per community health service centre/station, 6.13 all-age stroke YLDs per community health worker, and 16.7 all-age stroke YLDs per community health bed. Among adults aged 65 years or older, the corresponding descriptive ratios were 77.0 YLDs per community health service centre/station and 10.0 YLDs per community health bed. Relative to long-term-care denominators, there were 0.35 stroke YLDs among adults aged 65 years or older per elder-care bed and 0.46 stroke YLDs among adults aged 75 years or older per nursing-type registered elder-care bed. These ratios are contextual planning indicators only and do not quantify stroke-specific service access, adequacy, utilisation, quality, or unmet need (Table 4, Fig 4).

**Table 4:** Rehabilitation-relevant stroke burden in relation to community and long-term-care service capacity in China, 2023.

Capacity indicator	Stroke burden numerator	Resource denominator	Burden-to-capacity ratio
All-age stroke YLDs per community health service centre/station	4,766,612	37,177	128.2 YLDs per centre/station
Age 65 + stroke YLDs per community health service centre/station	2,862,419	37,177	77.0 YLDs per centre/station
All-age stroke YLDs per community health personnel	4,766,612	778,000	6.13 YLDs per person
Age 65 + stroke YLDs per community health personnel	2,862,419	778,000	3.68 YLDs per person
All-age stroke YLDs per community health bed	4,766,612	285,176	16.7 YLDs per bed
Age 65 + stroke YLDs per community health bed	2,862,419	285,176	10.0 YLDs per bed
Age 65 + stroke YLDs per elder-care bed	2,862,419	8,230,000	0.35 YLDs per bed
Age 75 + stroke YLDs per nursing-type registered elder-care bed	1,411,433	3,046,308	0.46 YLDs per nursing-type bed
Age 85 + stroke YLDs per nursing-type registered elder-care bed	377,807	3,046,308	0.12 YLDs per nursing-type bed

**Fig 4:**
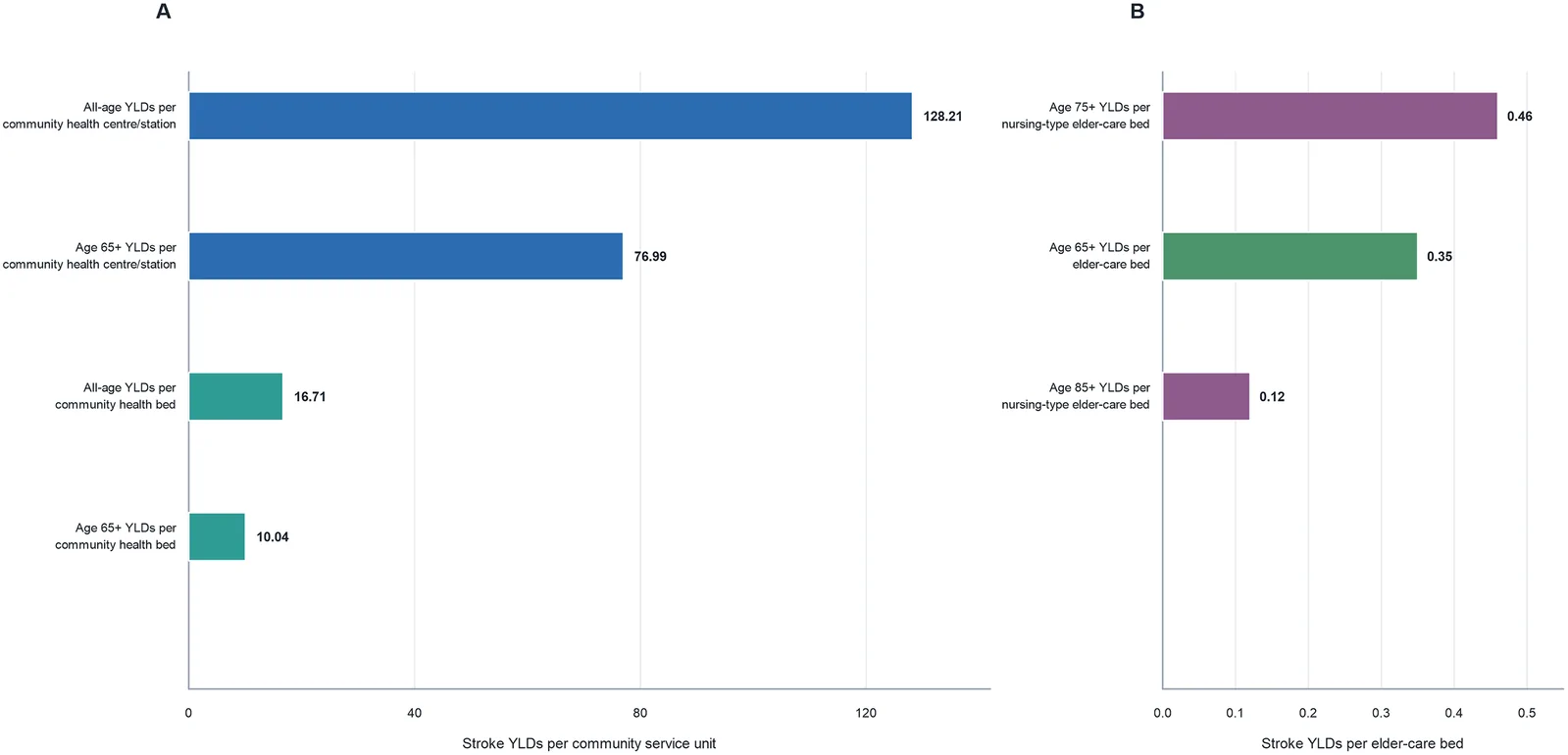
Rehabilitation-relevant stroke burden in relation to community and long-term-care service capacity in China, 2023.

Burden numerators are YLDs for ischaemic stroke plus intracerebral haemorrhage from GBD 2023. Resource denominators are national service-capacity indicators from official Chinese health and ageing statistics. Detailed denominator definitions are provided in S2 Table. Ratios are planning proxies and should not be interpreted as direct measures of rehabilitation access, utilisation, quality, or adequacy. Formal uncertainty propagation was not performed for these derived ratios, which are presented as descriptive point estimates.

Bars show descriptive YLD-based burden-to-capacity proxy indicators for combined ischaemic stroke and intracerebral haemorrhage in relation to selected community health and elder-care service denominators. The ratios provide planning context only and should not be interpreted as direct measures of rehabilitation access, adequacy, utilisation, quality, shortage, or unmet need. Point estimates are shown; formal uncertainty propagation was not performed.

## Discussion

This population-based analysis shows that stroke burden in China is increasingly shaped by disability, survivorship, and ageing. Four findings are central. First, the YLD share of stroke DALYs increased from 1990 to 2023, especially for ischaemic stroke. Second, EAPC analysis showed sustained increases in crude all-age YLD rate and YLD share, indicating that rehabilitation-relevant burden increased over time rather than appearing only as an endpoint difference. Third, burden shifted towards older-old and oldest-old adults, particularly those aged 75–84 years and 85 years or older. Fourth, placing stroke YLDs alongside selected national service-resource denominators provided descriptive planning context for community health and elder-care systems; these proxy indicators do not establish service adequacy, shortage, access, or unmet need.

The study extends previous Chinese GBD analyses of stroke burden [21–23] and related population-based work on stroke risk [24] in two ways. First, it focuses explicitly on the disability component of burden by examining YLDs and YLD share. This perspective is important because rehabilitation planning is more closely related to non-fatal disability than to mortality alone. Second, it places YLD estimates alongside national community health and elder-care service indicators to generate transparent, descriptive planning ratios. These ratios do not demonstrate service shortage, adequacy, or unmet need; rather, they make the scale of disability burden interpretable in relation to selected national capacity denominators.

The divergence between intracerebral haemorrhage and ischaemic stroke has practical implications. The crude all-age DALY rate for intracerebral haemorrhage declined, but crude rates are influenced by population age structure and should not be interpreted as age-independent changes in underlying epidemiological risk. By contrast, ischaemic stroke showed increasing crude all-age DALY and YLD rates, and its YLD share more than doubled. In this study, crude all-age rates were retained as population-level indicators relevant to service pressure, while age-specific subgroup analyses and decomposition were used to distinguish demographic pressure from age-specific rate change. The overall pattern indicates that progress in acute stroke care and prevention does not eliminate the need for long-term disability management. As more people survive ischaemic stroke, the cumulative burden relevant to rehabilitation, follow-up, secondary prevention, home support, and long-term functional care may continue to rise.

The oldest-old shift is especially important. Rehabilitation after stroke in adults aged 85 years or older is often complicated by frailty, sarcopenia, cognitive impairment, multimorbidity, pre-existing disability, and limited caregiver resources [5–7]. In this context, rehabilitation goals may include functional maintenance, prevention of avoidable decline, safe mobility, swallowing and nutrition support, caregiver training, prevention of complications, and coordination with long-term-care services. A burden profile that shifts towards the oldest-old therefore implies a different model of post-stroke care, one that combines neurological rehabilitation with geriatric assessment and community-based support [25–32].

The service-capacity proxy analysis should be interpreted cautiously and only as a descriptive planning lens. Community health services and elder-care facilities are not equivalent to specialised stroke rehabilitation services, and national denominators cannot capture regional differences, staffing mix, therapy intensity, referral pathways, utilisation, or service quality. The calculated ratios therefore do not demonstrate a service gap, shortage, adequacy, or unmet need. They simply place the national scale of stroke-related disability alongside selected community and age-care service-resource denominators. This interpretation is consistent with the Lancet Neurology Commission on Neurorehabilitation’s call for pragmatic, scalable, and context-responsive solutions that strengthen rehabilitation systems across the continuum of neurological care [33].

The decomposition analysis adds a systems-level explanation. For both subtypes, favourable age-specific DALY rate changes were large but were offset by population growth and ageing. This explains why health systems may experience sustained or rising absolute burden even when prevention and acute care improve. From a policy perspective, prevention, acute stroke systems, rehabilitation, community follow-up, and long-term-care coordination should be planned as complementary components rather than separate programmes.

This study has several strengths. It uses a long observation period from 1990 to 2023, examines two major stroke subtypes separately, focuses on YLDs and YLD share as rehabilitation-relevant indicators, evaluates older-age subgroups including the oldest-old, applies decomposition analysis, and links burden estimates with national service-capacity indicators. This integrated approach helps move beyond conventional burden description towards service-planning interpretation.

The study also has limitations. First, GBD estimates are modelled and depend on the quality, availability, and comparability of underlying data sources. Second, the analysis used national estimates and could not assess provincial, urban-rural, or socioeconomic variation, which is likely to be substantial in China. Third, only ischaemic stroke and intracerebral haemorrhage were included; subarachnoid haemorrhage was excluded because of its distinct aetiology and disability profile. Fourth, the age analysis used grouped age bands and selected comparison years; age-share estimates should be interpreted within the dataset for adults aged 50 years or older. Fifth, the reported all-age DALY and YLD rates are crude rates and are influenced by population age structure; they should not be interpreted as age-independent epidemiological trends. Age-specific subgroup analyses and decomposition were used to provide complementary information on demographic and age-specific rate contributions. Sixth, service-resource indicators were used as capacity proxies and do not measure stroke-specific rehabilitation availability, utilisation, referral pathways, staffing mix, therapy intensity, quality, cost, adequacy, or unmet need. Seventh, YLDs are based on modelled disability weights and do not capture individual functional status, rehabilitation eligibility, stroke severity, recurrence, cognitive impairment, dysphagia, or post-stroke care pathways. Eighth, formal uncertainty propagation was not performed for YLD share, decomposition components, or burden-to-capacity ratios because posterior draws and covariance information across GBD-derived quantities were unavailable in the exported dataset; these derived indicators should therefore be interpreted descriptively as point estimates. Finally, decomposition provides an analytical explanation of burden change but should not be interpreted as direct causal evidence.

Future research should link burden estimates with province-level rehabilitation resources, urban-rural service distribution, workforce data, stroke rehabilitation utilisation, functional outcome registries, and long-term-care use. Such work could determine whether areas with higher stroke disability burden also experience mismatches in rehabilitation resources and whether community-based rehabilitation and elder-care services are aligned with the needs of older stroke survivors.

## Conclusions

Stroke in China is increasingly characterised by long-term disability, survivorship, and concentration among older-old and oldest-old adults. Crude all-age rates, age-specific analyses, and decomposition together show that population ageing is integral to the observed burden pattern. Placing YLD estimates alongside selected community health and elder-care denominators provides descriptive planning context, but the resulting proxy indicators do not measure rehabilitation access, service adequacy, quality, shortage, or unmet need. The findings support coordinated planning across stroke prevention, acute care, community rehabilitation, geriatric care, caregiver support, and long-term-care services for older stroke survivors.

## Supporting information

S1 Table GBD cause definitions and ICD reference mappings for stroke subtype extraction. (DOCX)

S2 Table Source definitions for service-capacity denominators used in Table 4. (DOCX)

S1 Fig Age-specific DALY rates for ischaemic stroke in China, selected years. Lines show age-specific DALY rates per 100,000 population among adults aged 50 years or older in selected years from 1990 to 2023.
(TIF)

S2 Fig Age-specific DALY rates for intracerebral haemorrhage in China, selected years. Lines show age-specific DALY rates per 100,000 population among adults aged 50 years or older in selected years from 1990 to 2023.
(TIF)

S3 Fig Divergent crude all-age DALY rate trends by stroke subtype in China, 1990–2023. Lines show crude all-age DALY rates per 100,000 population for ischaemic stroke and intracerebral haemorrhage. Point estimates are shown.
(TIF)

S1 Data Extracted analytical dataset used to reproduce the tables and figures. (XLSX)

S2 Data CSV archive containing the machine-readable source and derived datasets used by S1 Code. (ZIP)

S1 Code Python code used to reproduce the EAPC, decomposition, figure-data, and service-capacity proxy calculations. (PY)
