# Supplementary material for: Stroke disability burden and rehabilitation service-capacity proxies in China, 1990–2023: A population-based analysis using GBD 2023 and national service statistics
Source: PLoS One. 2026 Nov 6;21(11):e0361758. doi: 10.1371/journal.pone.0361758 (PMC13743016; doi:10.1371/journal.pone.0361758)
Supplement: Supplementary file 1 [file pone.0361758.s001.docx]

**S1 Table. GBD cause definitions and ICD reference mappings for stroke subtype extraction.**

| **Stroke subtype** | **GBD cause name used in Results Tool** | **Primary extraction standard** | **GBD hierarchy** | **Core clinical definition** | **ICD-10 reference mapping*** | **ICD-9 reference mapping*** | **Included in present analysis** | **Notes** |
| --- | --- | --- | --- | --- | --- | --- | --- | --- |
| Ischaemic stroke | Ischemic stroke | Predefined GBD cause name | Level 3 stroke subtype under cerebrovascular disease | Cerebral infarction caused by arterial occlusion | I63–I63.9 | 433–434, 436 | Yes | Data were extracted directly by GBD cause name; ICD mappings are shown for reference only |
| Intracerebral haemorrhage | Intracerebral hemorrhage | Predefined GBD cause name | Level 3 stroke subtype under cerebrovascular disease | Non-traumatic intraparenchymal brain haemorrhage | I61–I61.9 | 431 | Yes | Data were extracted directly by GBD cause name; ICD mappings are shown for reference only |

Abbreviations: GBD, Global Burden of Disease; ICD, International Classification of Diseases. The study used the predefined GBD cause names “Ischemic stroke” and “Intracerebral hemorrhage” in the IHME GBD Results Tool as the primary extraction standard. ICD-9 and ICD-10 codes are provided as reference mappings only; final GBD cause assignment is based on the official cause list, cause metadata, and redistribution procedures.
