# Supplementary material for: Stroke disability burden and rehabilitation service-capacity proxies in China, 1990–2023: A population-based analysis using GBD 2023 and national service statistics
Source: PLoS One. 2026 Nov 6;21(11):e0361758. doi: 10.1371/journal.pone.0361758 (PMC13743016; doi:10.1371/journal.pone.0361758)
Supplement: Supplementary file 2 [file pone.0361758.s002.docx]

**S2 Table. Source definitions for service-capacity denominators used in Table 4.**

| **Denominator** | **Value** | **Definition and calculation** | **Exact source** | **Notes** |
| --- | --- | --- | --- | --- |
| Community health service centres/stations | 37,177 | Community health service centres (10,070) plus community health service stations (27,107) at year-end 2023. | Refs 18 and 20 (URLs and accessed dates in reference list). China Health Statistics Yearbook 2024, Table 6-2 'Community health service institutions, beds and personnel' and Table 1-1-1 'Number of health care institutions'; Statistical Bulletin on China's Health Development in 2023, Table 1 'National health care institutions and beds'. | The total equals 10,070 + 27,107; no additional aggregation was applied. |
| Community health personnel | 778,000 | Personnel in community health service centres/stations. The ratio table uses the public-bulletin rounded value of 77.8 ten-thousand persons. | Refs 18 and 20 (URLs and accessed dates in reference list). Statistical Bulletin on China's Health Development in 2023, primary-care services section and Table 3 'Personnel in health care institutions by category'; China Health Statistics Yearbook 2024, Table 6-2 and Table 2-6-1 'Personnel in community health service centres/stations by region'. | The exact yearbook value is 777,594 persons; 778,000 is the rounded value used for the denominator. |
| Community health beds | 285,176 | Beds in community health service centres/stations, calculated as community health service centre beds (274,193) plus community health service station beds (10,983). | Ref 20 (URL and accessed date in reference list). China Health Statistics Yearbook 2024, Table 6-2 'Community health service institutions, beds and personnel'; Table 3-1-2 'Beds in health care institutions by institution type'; and Table 3-3 'Beds in primary health care institutions'. | The public bulletin reports 274,193 beds for community health service centres and 10,983 beds for community health service stations separately. The approximately 27.4 ten-thousand beds figure refers only to community health service centres and is a rounded bulletin value; the denominator used here is the exact centre+station yearbook total. |
| Elder-care beds | 8,230,000 | All elder-care beds in elder-care institutions and facilities at year-end 2023. | Ref 19 (URLs and accessed date in reference list). National Bulletin on the Development of Ageing in 2023, section 'Elder-care service system'. | The bulletin reports 8.23 million total elder-care beds, including 5.172 million beds in registered elder-care institutions and 3.058 million beds in community elder-care service institutions/facilities. |
| Nursing-type registered elder-care beds | 3,046,308 | Registered elder-care institution beds (5,172,000) multiplied by the reported proportion of nursing-type beds (58.9%). | Ref 19 (URLs and accessed date in reference list). National Bulletin on the Development of Ageing in 2023, section 'Elder-care service system'. | Calculation: 5,172,000 x 0.589 = 3,046,308. This derived count applies only to registered elder-care institution beds because the reported 58.9% nursing-type proportion is stated for that subgroup. |

These denominators are national service-capacity indicators used only as descriptive planning proxies. They do not measure stroke-specific rehabilitation access, staffing mix, therapy intensity, utilisation, quality, adequacy, or unmet need.
